# Changes in upper limb kinematics in children with cerebral palsy after lower limb surgery: a retrospective comparative study

**DOI:** 10.1186/s12891-026-09692-2

**Published:** 2026-03-03

**Authors:** Yasar Samet Gokceoglu, Fuat Bilgili, Cansu Sardogan, Daghan Koyuncu, Mehmet Demirel, Ekin Akalan

**Affiliations:** 1 Department of Orthopedics and Traumatology, University of Health Sciences Mehmet Akif Inan Research and Training Hospital, Sanliurfa, Turkey; 2 Department of Orthopedics and Traumatology, Istanbul Faculty of Medicine, Istanbul University, Istanbul, Turkey; 3 Istanbul Faculty of Medicine, Physical Therapy and Rehabilitation, İstanbul University, İstanbul, Turkey

**Keywords:** Gait analysis, Diplegia, Hemiplegia, Cerebral palsy, Kinovea

## Abstract

**Background:**

Cerebral palsy (CP), which is characterized by movement and posture disorders, is a neurological disorder that affects the movement of both the lower and upper extremities. Current research on gait analysis in children with cerebral palsy is mostly focused on the lower extremities, whereas research on the upper extremities is limited to three-dimensional gait analysis. However, in many countries, video-based gait analysis is used instead of three-dimensional gait analysis owing to its high cost. The primary aim of this study was to determine whether upper extremity kinematics change after lower extremity surgery in children with cerebral palsy. As a secondary aim, we developed a video-based analysis method to assess these changes in settings where 3D gait analysis is not available.

**Methods:**

The study included 30 children (17 diplegic and 13 hemiplegic including 18 boys and 12 girls) with a mean age of 8.9 years and 29 healthy children (15 boys and 14 girls) with a mean age of 9.3 years. The efficacy of the surgical procedure was determined using Edinburgh Visual Gait Scores before and after surgery. Bilateral upper limb kinematics, including wrist, elbow, shoulder, trunk, and head flexion/extension angles, as well as trunk and head lateral flexion and shoulder abduction in the coronal plane, were measured during the initial contact and mid-stance phases using Kinovea 0.9.5 software by 2 different observers. Measurements from the more and less affected sides in the CP group were compared with corresponding limbs of the healthy control group. Preoperative, postoperative, and control groups were compared by ANOVA. The ICC test was used to evaluate the interobserver reliability between the 2 observers. Wilcoxon signed-rank test was used to compare EVGS scores.

**Results:**

Postoperative outcomes at the wrist and elbow were found to differ in the sagittal plane, with notable adjustments in the flexion/extension angles during the initial contact and midstance phases. In addition, compared with those in the preoperative phase, the Edinburgh Scale (Edinburgh) score showed favorable postoperative improvements. Postoperative improvements were observed in wrist and elbow kinematics, with values approaching those of the control group. Interobserver reliability was excellent for most sagittal plane measurements (ICC > 0.90).

**Conclusions:**

This study revealed changes in the kinematics of the upper extremities after lower extremity surgery. These findings demonstrates that video-based analysis can provide reliable measurements of upper limb kinematics in settings where 3D gait analysis is not available.

**Trial registration:**

This study was registered with ClinicalTrials.gov, number NCT05957783, titled “Changes in Upper Limb Kinematics in Children with Cerebral Palsy After Lower Limb Surgery.” The trial was publicly registered on 07/24/2023 to ensure transparency and reproducibility of the research process. Although the data were analyzed retrospectively, the study protocol was prospectively registered at ClinicalTrials.gov (NCT05957783) to ensure transparency. The party responsible for the trial registration was Yaşar Samet Gökçeoğlu, which is contactable via sametgokceoglu@gmail.com.

 Cerebral palsy (CP) is the most common cause of childhood physical disability. As defined by the Surveillance of Cerebral Palsy in Europe (SCPE) and subsequent key consensus statements, CP describes a group of permanent disorders of movement and posture causing activity limitation, attributed to non-progressive disturbances occurring in the developing fetal or infant brain [1–3]. These disorders are often accompanied by disturbances of sensation, perception, cognition, communication, and behavior, by epilepsy, and by secondary musculoskeletal problems [4]. Loss of motor function and gait disturbances can affect the lower and upper extremities and can be classified as diplegic, hemiplegic, or quadriplegic, depending on the location of the injury in the brain [5]. These children are evaluated in gait analysis laboratories for gait, lower extremity, and upper extremity kinematics [6].

A review of the literature describing upper extremity movement during gait and gait disorders showed that upper extremity movement facilitates lower extremity movement and reduces energy expenditure by 8% [7]. In pathological gait, especially in neurological disorders such as CP, Parkinson’s disease, and stroke, upper extremity function is usually compromised, as evidenced by data measured in gait analysis laboratories [8].

Gait analysis laboratories performed video-based, kinetic, kinematic, and 3D gait analyses. Three-dimensional (3D) gait analysis is the gold standard for gait assessment. However, in underdeveloped or semideveloped countries, video-based gait analysis is performed because Three-Dimensional (3D) gait analysis is expensive [9]. A study comparing human performance analysis laboratories in the Turkish population with those in the American population revealed that gait analysis laboratories were less common [10]. In an international survey of gait analysis laboratories, only 44% of the laboratories included in the international survey examined the upper extremities, and 33% performed 3D analysis [11].

In the current literature, while there are studies examining upper limb function in CP using various methods, research specifically focusing on upper extremity kinematics during gait using video-based analysis is notably limited [11]. Most existing studies evaluate upper extremities through functional scales and task-based assessments, but comprehensive analyses of upper extremity kinematics during gait using video-based analysis methods are lacking. Our primary hypothesis is that lower extremity surgery leads to significant changes in upper extremity kinematics in children with diplegic or hemiplegic CP. To test this hypothesis, we compared upper extremity kinematics before and after lower extremity surgery, using a video-based analysis method as a practical alternative to Three-Dimensional (3D) gait analysis. The development of such videos is secondary, let us say of ‘by-product’ importance, our main concern was how the movements of arms during walking are affected by the surgical correction of lower limb deformities. The method of the video-gait analysis utilized in this study was able to document considerable alterations in upper limb performance following lower limb rehabilitation, thus it was a useful and practical substitute in places where 3D gait analysis was unavailable.

## Methods

### Study design and setting

This was a retrospective study analyzing gait laboratory records of children with CP who underwent lower extremity surgery between 2018 and 2023 at Istanbul University Medical Faculty. Control group data was collected prospectively from healthy volunteers during the same period.

### Participants and selection

From the gait analysis laboratory archive of Istanbul University Medical Faculty, 300 CP patients who had lower extremity surgery were initially screened. After applying all inclusion and exclusion criteria, 30 patients were eligible for the study. The inclusion criteria were: (1) confirmed diagnosis of diplegic or hemiplegic CP, (2) underwent lower extremity orthopedic surgery, (3) availability of both pre- and post-operative gait analysis records, (4) no previous upper extremity surgery, (5) no upper extremity contracture. The exclusion criteria were: (1) incomplete video records, (2) assisted walking in videos, (3) poor video quality, (4) incompatible video format with Kinovea software, (5) use of orthotics during walking. From the initially screened 300 patients, 270 were excluded due to one or more of these criteria. Thirty patients with CP underwent orthopaedic procedures, including achilloplasty (*n* = 5), varization derotation osteotomy (*n* = 8), periacetabular osteotomy (*n* = 3), gastrocnemius release (*n* = 20), hamstring release (*n* = 22), and midfoot osteotomies (*n* = 2). The inclusion criteria were confirmed diagnosis of diplegic or hemiplegic CP, lower extremity orthopedic surgery history, availability of both pre- and post-operative gait analysis records, no previous upper extremity surgery, and no upper extremity contracture.

The high exclusion rate (270 out of 300 patients) was primarily due to technical limitations inherent to the retrospective design. The most frequent reasons for exclusion were poor video quality, incomplete preoperative or postoperative recordings, incompatible file formats with the Kinovea software, assisted walking during video recording, and the use of orthotics during gait. These strict criteria were necessary to ensure that only high-quality, analyzable videos were included, thereby minimizing measurement errors.

### Data collection and equipment

Video recordings were performed using a high-definition Nikon Coolpix S100 camera (60 frames/second) mounted on a tripod. The setup included a 10-meter walkway with the camera positioned perpendicular to the walking direction at its midpoint, 3 m from the walkway centerline. During video recordings, participants walked barefoot and wore form-fitting shorts and sleeveless tops to ensure clear visualization of joint movements while maintaining participant dignity. Anatomical markers were not used due to the retrospective nature of the study, which is acknowledged as a limitation.

For the reliability analysis, ten patients were randomly selected from the eligible CP group. Their preoperative gait videos were analyzed independently by two observers (YSG and FB) using the same Kinovea 0.9.5 setup described above. Both observers measured all defined sagittal and coronal plane joint angles for the initial contact and mid-stance phases. Observers were blinded to each other’s results to prevent bias.

### Kinematic analysis

For standardization, all measurements were performed using Kinovea software version 0.9.5. Joint angles were measured in both sagittal and coronal planes using specific anatomical landmarks as reference points.

Sagittal plane measurements:

1. Wrist flexion/extension: Measured between the hand segment and forearm segment, using the lateral wrist joint as the vertex.
2. Elbow flexion/extension: Measured between the forearm segment and upper arm segment, using the lateral epicondyle as the vertex.
3. Shoulder flexion/extension: Measured between the upper arm segment and the vertical trunk line, using the lateral shoulder joint as the vertex.

Coronal plane measurements:

1. Shoulder abduction/adduction: Measured between the upper arm segment and the vertical trunk line, using the acromion process as the reference point.
2. Trunk lateral flexion: Measured between the C7 vertebra to sacrum line and the global vertical line.

**Fig. 1:**
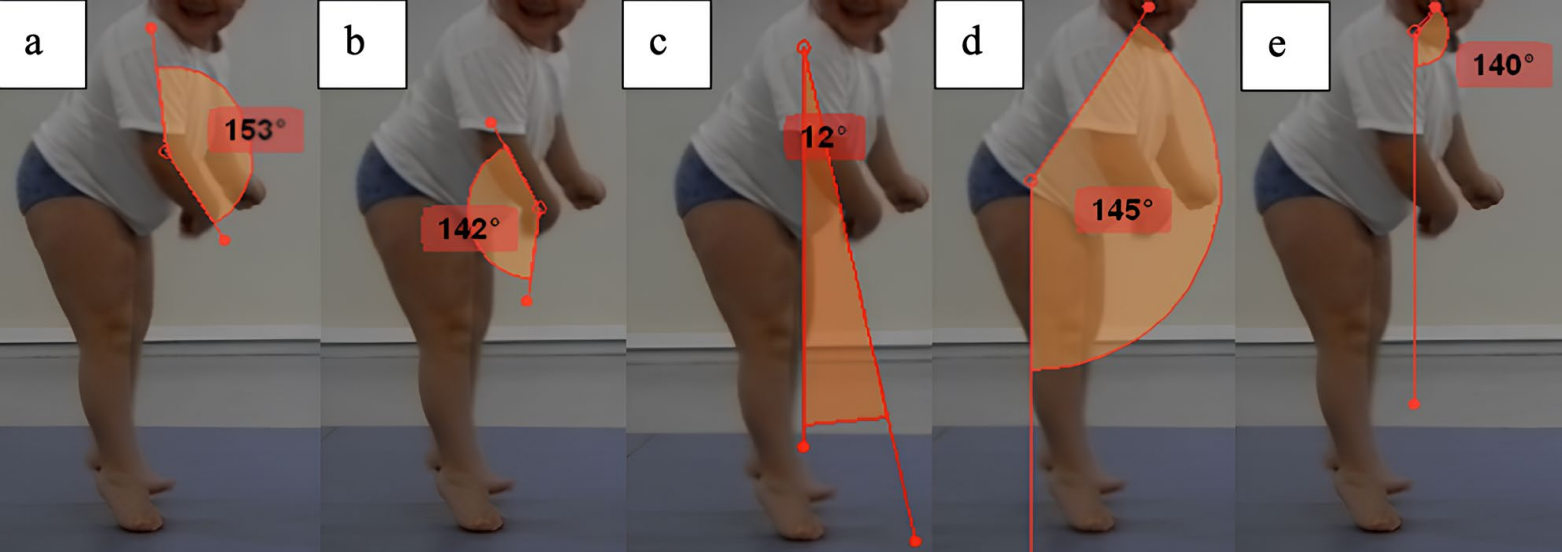
Upper extremity measurements in the sagittal plane during the midstance phase. (**a)** elbow sagittal plane angle, (**b**) wrist sagittal plane angle, (**c**) shoulder sagittal plane angle, (**c**) trunk sagittal plane angle, and (**d**) head sagittal plane angle

**Fig. 2:**
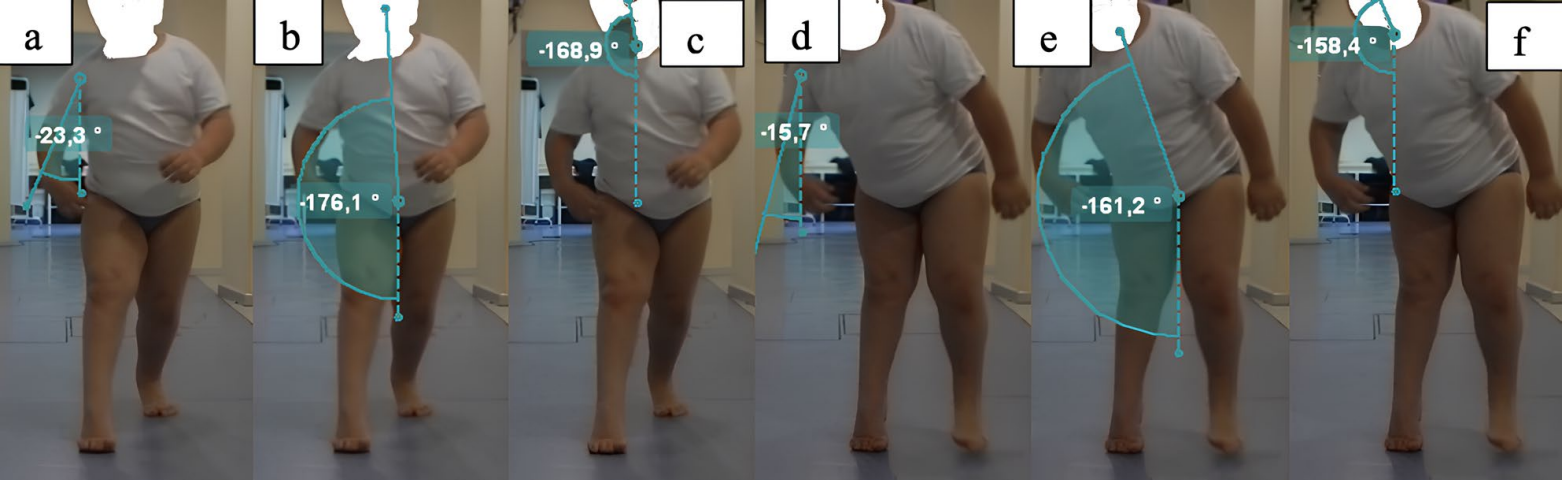
Coronal plane upper extremity measurements. (**a)** Initial contact shoulder coronal plane angle, (**b**) initial contact trunk coronal plane angle, (c) initial contact head coronal plane angle, (**c**) midstance shoulder coronal plane angle, (**d**) midstance trunk coronal plane angle, and (**f**) midstance head coronal plane angle

The gait cycle was divided into eight standardized phases: initial contact, loading, mid-stance, terminal stance, preswing, early swing, mid-swing, and terminal swing. Measurements were performed at each phase using the defined reference points and lines (Figs. 1 and 2,).

#### Side designation and analysis

Patients with cerebral palsy (CP) were classified clinically into diplegic or hemiplegic subtypes. For the purposes of kinematic analysis, each patient’s limbs were further designated as ‘more affected’ or ‘less affected’ based on clinical presentation and gait analysis findings. In hemiplegic patients, the involved limb was designated as the ‘more affected’ side. In diplegic patients with asymmetric involvement, the side with greater functional impairment was designated as ‘more affected’; in symmetric cases, side designation was randomized.

Statistical analyses were performed using these limb-based categories (more affected vs. less affected sides) and compared to the corresponding limbs of healthy control participants. Pre- and postoperative measurements were analyzed within the CP group, and subgroup analyses for diplegic and hemiplegic patients were conducted where relevant.

#### EVGS assessment

The Edinburgh Visual Gait Score (EVGS), a validated tool for video-based gait analysis in CP [9, 14], was used to evaluate gait. The EVGS has shown good reliability in previous studies [9, 14] and includes 17 items for gait assessment. EVGS scoring was performed by an expert physiotherapist (CS).

### Statistical analysis

#### Sample size and power analysis

Sample size and power calculations were performed using eta-squared test. The eta-squared value of 0.14 was set as the threshold for large effect size based on previous literature [12]. These calculations were performed prior to the main statistical analyses to ensure adequate sample size for detecting clinically meaningful differences.

#### Reliability analysis

For the reliability analysis, ten patients were randomly selected from the eligible CP group. Their gait videos were analyzed independently by two observers (YSG and FB), each performing the measurements once for all defined sagittal and coronal plane joint angles.

Interobserver reliability was calculated using the intraclass correlation coefficient (ICC), applying a two-way random-effects model with absolute agreement for single measurements (ICC [1, 2]). In this model, both the subjects and the raters are considered random samples from a larger population, and the absolute agreement definition accounts for any systematic differences between raters. ICC values were interpreted according to Koo and Li’s guidelines [13, 14]: excellent (> 0.90), good (0.75–0.90), moderate (0.50–0.75), and poor (< 0.50).

#### Comparative analysis

The primary comparison was between more affected and less affected limbs of patients with CP and corresponding limbs of healthy controls. Additional comparisons included pre- vs. postoperative measurements within the patient group and subgroup analyses stratified by CP type (diplegic vs. hemiplegic). This approach ensured that both limb-specific and diagnosis-specific differences could be evaluated. Data normality was assessed using Shapiro-Wilk test. Three types of comparisons were performed for kinematic parameters:

- Three-group comparisons using one-way ANOVA with post-hoc Tukey test for normally distributed data and Kruskal-Wallis test with post-hoc Dunn analysis for non-normally distributed data.
- Pre versus post-operative comparisons within the same group using paired t-test for normally distributed data and Wilcoxon signed-rank test for non-normally distributed data.
- Between-group comparisons using independent t-test for normally distributed data and Mann-Whitney U test for non-normally distributed data.

EVGS comparisons were performed using Wilcoxon signed-rank test, as it is an ordinal scale measure. Categorical data were compared using chi-square test. Statistical significance was set at *p* < 0.05. All analyses were performed using SPSS version 21.0. All measurements were performed by two independent observers (YSG and FB) for reliability assessment.

## Results

### Demographic and clinical characteristics

The study included 30 patients with cerebral palsy (CP), of whom 17 were diplegic and 13 were hemiplegic, and a control group of 29 healthy individuals. The mean follow-up duration for the patient group was 3.2 ± 1.5 years post-surgery. The mean age was comparable between the groups, with 8.9 ± 2.56 years in the patient group and 9.3 ± 3.2 years in the control group (*P* = 0.75). Gender distribution was also similar between the groups, with 18 boys and 12 girls in the patient group and 15 boys and 14 girls in the control group (*P* = 0.1) (Table 1).

**Table 1:** Demographic Data

Category	Diplegic CP (*n* = 17)	Hemiplegic CP (*n* = 13)	Total CP (*n* = 30)	Control (*n* = 29)
Age (mean ± SD, years)	8.8 ± 2.4	9.0 ± 2.7	8.9 ± 2.56	9.3 ± 3.2
Gender (boys/girls)	10 / 7	8 / 5	18 / 12	15 / 14
Follow-up (mean ± SD, years)	3.3 ± 1.4	3.1 ± 1.6	3.2 ± 1.5	—
Operation type				
Iliopsoas release	6	4	10	—
Gastrocnemius muscle release (Vulpius)	12	8	20	—
Hamstring muscle release	14	8	22	—
Hip adductor release	5	2	7	—
Achilloplasty	3	2	5	—
Femur derotation osteotomy	5	3	8	—
Distal rectus femoris tendon transfer	4	2	6	—
Tibialis posterior Z-plasty	3	1	4	—
Foot osteotomies (forefoot, midfoot, hindfoot)	1	1	2	—

*SD* Standard Deviation, *CP* Cerebral Palsy

### Kinematic analysis

In children with diplegic or hemiplegic cerebral palsy who underwent lower-extremity surgery, gait analysis revealed significant changes in upper extremity kinematics during specific gait phases, including initial contact, midstance, terminal stance, initial swing, and terminal swing. Postoperative measurements demonstrated improvements on both the more affected and less affected sides, with values approaching those of the control group (Tables 2 and 3). All sagittal and coronal plane angles presented in Figs. 1 and 2 were measured and included in the reliability analysis (Table 4). Kinematic analyses were also performed for all these angles; however, due to the large volume of data, only clinically relevant parameters are presented in Tables 2 and 3.

**Table 2:** Comparison of upper extremity kinematics during initial contact and midstance phases in the sagittal plane. Values are presented as mean ± SD (degrees). Statistically significant p-values are marked with an asterisk (*)

Phase & Parameter	Pre-op	Post-op	Control	Pre vs. Post	Pre vs. Control	Post vs. Control
Initial Contact						
Wrist (MA*)	7.37 ± 11.39	8.43 ± 16.6	0.48 ± 1.5	0.797	0.012*	0.245
Elbow (MA*)	55.13 ± 30.93	28.87 ± 13.18	12.59 ± 9.06	0.037*	0.01*	0.365
Wrist (LA)	5.73 ± 13.54	13.7 ± 23.08	0.48 ± 1.5	0.735	0.345	0.234
Elbow (LA)	43.73 ± 35.95	14.7 ± 10.03	12.59 ± 9.06	0.041*	0.02*	0.456
Midstance						
Wrist (MA*)	28.13 ± 16.39	3.37 ± 4.63	0.72 ± 2.86	0.032*	0.004*	0.345
Elbow (MA*)	54.87 ± 21.25	20.53 ± 11.8	19.86 ± 9.54	0.001*	0.004*	0.365
Elbow (LA)	53.50 ± 33.57	27.23 ± 27.92	19.86 ± 9.54	0.002*	0.004*	0.365

*MA* More affected, *LA* Less affected, *SD* Standard Deviation, *Pre-op* Preoperative, *Post-op* Postoperative; *Pre vs. Post* Comparison between preoperative and postoperative values in the same group, *Pre vs. Control* Comparison between preoperative values in the patient group and control group values, *Post vs. Control* Comparison between postoperative values in the patient group and control group values

**Table 3:** Comparison of upper extremity kinematics during preswing, initial swing, midswing, and terminal swing phases in the sagittal plane. Values are presented as mean ± SD (degrees). Statistically significant *p*-values are marked with an asterisk (*)

Phase & Parameter	Pre-op	Post-op	Control	Pre vs. Post	Pre vs. Control	Post vs. Control
Preswing						
Wrist (MA)	9.0 ± 15.69	3.77 ± 13.7	0.34 ± 1.39	0.567	0.012*	0.245
Elbow (MA)	59.13 ± 27.04	54.0 ± 29.12	26.62 ± 13.59	0.328	0.045*	0.345
Wrist (LA)	5.83 ± 9.89	1.77 ± 1.44	0.34 ± 1.39	0.356	0.067	0.123
Elbow (LA)	61.63 ± 33.2	56.6 ± 28.68	26.62 ± 13.59	0.690	0.051	0.178
Initial Swing						
Wrist (MA)	27.73 ± 14.31	2.27 ± 1.44	0.1 ± 0.55	0.017*	0.021*	0.158
Elbow (MA)	57.9 ± 30.05	50.6 ± 29.31	20.0 ± 14.51	0.532	0.001*	0.258
Wrist (LA)	27.1 ± 11.73	1.73 ± 17.6	0.1 ± 0.55	0.024*	0.045*	0.198
Elbow (LA)	56.3 ± 32.65	54.2 ± 30.84	20.0 ± 14.51	0.412	0.035*	0.201
Midswing						
Wrist (MA)	7.4 ± 14.75	9.5 ± 19.27	0.59 ± 3.15	0.074	0.051	0.165
Elbow (MA)	60.07 ± 28.48	52.27 ± 32.29	17.59 ± 11.36	0.083	0.045*	0.321
Wrist (LA)	4.4 ± 8.57	8.2 ± 17.02	0.59 ± 3.15	0.084	0.051	0.201
Elbow (LA)	51.97 ± 33.79	47.0 ± 32.86	17.59 ± 11.36	0.837	0.063	0.125
Terminal Swing						
Wrist (MA)	28.67 ± 17.47	3.07 ± 5.76	0.31 ± 1.67	0.035*	0.001*	0.123
Elbow (MA)	58.13 ± 30.26	49.73 ± 28.87	13.76 ± 9.14	0.094	0.015*	0.175
Wrist (LA)	26.13 ± 19.13	2.43 ± 1.0	0.31 ± 1.67	0.028*	0.041*	0.145
Elbow (LA)	40.73 ± 36.75	42.43 ± 34.86	13.76 ± 9.14	0.404	0.031*	0.187

*MA* More affected, *LA* Less affected, *SD* Standard Deviation, *Pre-op* Preoperative, *Post-op* Postoperative, *Pre vs. Post* Comparison between preoperative and postoperative values in the same group; *Pre vs. Control* Comparison between preoperative values in the patient group and control group values, *Post vs. Control *Comparison between postoperative values in the patient group and control group values

**Table 4:** Interobserver reliability of upper extremity kinematics values are presented as intraclass correlation coefficient (icc) with. *95% Confidence Intervals (C.I.)*

Phase & Parameter	ICC	C.I. 95%	Phase & Parameter	ICC	C.I. 95%
Initial Contac					
Wrist (MA)	0.916	[0.837; 0.961]	Wrist (H)	0.910	[0.817; 0.956]
Elbow (MA)	0.923	[0.856; 0.966]	Elbow (H)	0.987	[0.972; 0.993]
Shoulder (MA)	0.905	[0.824; 0.956]	Shoulder (H)	0.895	[0.789; 0.949]
Trunk	0.654	[0.417; 0.833]	Head	0.786	[0.593; 0.893]
Preswing					
Wrist (MA)	0.997	[0.993; 0.998]	Wrist (H)	0.994	[0.987; 0.997]
Elbow (MA)	0.991	[0.981; 0.995]	Elbow (H)	0.999	[0.997; 0.999]
Shoulder (MA)	0.940	[0.876; 0.971]	Shoulder (H)	0.906	[0.810; 0.954]
Trunk	0.806	[0.628; 0.903]	Head	0.925	[0.847; 0.964]
Midstance					
Wrist (MA)	0.992	[0.983; 0.996]	Wrist (H)	0.988	[0.974; 0.994]
Elbow (MA)	0.976	[0.949; 0.988]	Elbow (H)	0.993	[0.985; 0.996]
Shoulder (MA)	0.932	[0.860; 0.967]	Shoulder (H)	0.923	[0.843; 0.963]
Trunk	0.726	[0.495; 0.861]	Head	0.880	[0.761; 0.941]
Terminal Stance					
Wrist (MA)	0.972	[0.941; 0.986]	Wrist (H)	0.991	[0.981; 0.995]
Elbow (MA)	0.985	[0.968; 0.992]	Elbow (H)	0.993	[0.985; 0.996]
Shoulder (Ma)	0.915	[0.827; 0.959]	Shoulder (H)	0.932	[0.860; 0.967]
Trunk	0.879	[0.759; 0.941]	Head	0.907	[0.812; 0.955]
Terminal Swing					
Wrist (MA)	0.992	[0.983; 0.996]	Wrist (H)	0.991	[0.981; 0.995]
Elbow (MA)	0.985	[0.968; 0.992]	Elbow (H)	0.990	[0.978; 0.995]
Shoulder (MA)	0.972	[0.941; 0.986]	Shoulder (H)	0.896	[0.791; 0.949]
Trunk	0.865	[0.733; 0.934]	Head	0.912	[0.821; 0.957]

*MA* More affected, *LA* Less affected, *ICC* Intraclass Correlation Coefficient, *CI* Confidence Intervals

#### Stance phase changes

During the initial contact phase, significant improvements were observed in elbow angles. On the more affected side, wrist angle showed minimal change (7.37 ± 11.39° to 8.43 ± 16.6°; *P* = 0.797), but preoperative wrist values differed significantly from controls (*P = 0.012). Elbow angle improved significantly, decreasing from 55.13 ± 30.93° to 28.87 ± 13.18° (*P* = 0.037). Preoperative elbow values differed significantly from controls (*P* = 0.01), while postoperative values showed no significant difference (*P* = 0.365). On the less affected side, wrist angle changes were not statistically significant (5.73 ± 13.54° to 13.7 ± 23.08°; *P* = 0.735). However, elbow angles improved significantly, decreasing from 43.73 ± 35.95° to 14.7 ± 10.03° (*P* = 0.041), with preoperative values differing significantly from controls (*P* = 0.02) but not postoperatively (*P* = 0.456). During the midstance phase, wrist and elbow angles showed significant improvement. On the more affected side, wrist angle decreased significantly from 28.13 ± 16.39° to 3.37 ± 4.63° (*P* = 0.032), with preoperative values differing from controls (*P* = 0.004). Postoperative wrist values were no longer significantly different from controls (*P* = 0.345). Similarly, elbow angle decreased markedly from 54.87 ± 21.25° to 20.53 ± 11.8° (*P* = 0.001), again demonstrating significant preoperative differences from controls (*P* = 0.004) but no difference postoperatively (*P* = 0.365). On the less affected side, elbow angles also improved significantly, decreasing from 53.50 ± 33.57° to 27.23 ± 27.92° (*P* = 0.002), showing a similar pattern of significant preoperative difference from controls (*P* = 0.004) but no postoperative difference (*P* = 0.365) (Table 2).

#### Reliability analysis

Interobserver reliability analysis was conducted on measurements from ten patients. The analysis demonstrated excellent reliability (*ICC > 0.90*) for most sagittal plane measurements of wrist and elbow angles across all gait phases. During the initial contact phase, wrist (ICC = 0.916; CI: 0.837–0.961) and elbow (ICC = 0.923; CI: 0.856–0.966) measurements showed excellent reliability on the more affected side, while on the less affected side, wrist (ICC = 0.910; CI: 0.817–0.956) and elbow (ICC = 0.987; CI: 0.972–0.993) displayed similarly high reliability. The midstance phase demonstrated the highest reliability values for wrist measurements, with ICC values reaching 0.992 (CI: 0.983–0.996) on the more affected side and 0.988 (CI: 0.974–0.994) on the less affected side. For shoulder measurements, good to excellent reliability was observed. Sagittal plane measurements (ICC range: 0.895–0.932) performed slightly better than coronal plane measurements (ICC range: 0.854–0.917). Reliability was comparatively lower for trunk measurements, which showed moderate reliability (ICC = 0.726; CI: 0.495–0.861). In contrast, head measurements demonstrated good reliability with an ICC value of 0.880 (CI: 0.761–0.941) (Table 5).

**Table 5:** Comparison of Edinburgh Visual Gait Scores (EVGS) between preoperative and postoperative phases. Values are presented as mean ± SD. Statistically significant *p*-values are marked with an asterisk ().*

Group & Phase	*N*	Min	Max	Mean ± SD	*P*-value
More Affected Side					
Preoperative	30	6	25	17.97 ± 5.15	0.006*
Postoperative	30	6	21	12.97 ± 3.85	
Less Affected Side					
Preoperative	30	8	29	16.73 ± 5.45	0.01*
Postoperative	30	8	22	13.00 ± 3.56	

*SD* Standard Deviation

### Edinburgh visual gait score analysis

The Edinburgh Visual Gait Score (EVGS) demonstrated significant improvements following surgery on both the more affected and less affected sides. On the more affected side, the EVGS decreased significantly from 17.97 ± 5.15 preoperatively to 12.97 ± 3.85 postoperatively (*P* = 0.006). On the less affected side, the EVGS also improved significantly, decreasing from 16.73 ± 5.45 preoperatively to 13.00 ± 3.56 postoperatively (*P* = 0.01). These findings indicate a clear improvement in overall gait patterns following surgery (Table 5).

## Discussion

The primary finding of our study was that upper extremity kinematics during gait showed significant improvements after lower extremity surgery in children with spastic CP, with measurements approaching those of healthy controls. This improvement was observed particularly in wrist and elbow kinematics during specific gait phases. The differential response between affected and less affected sides, and the post-surgical changes in upper limb movement patterns, can be attributed to several factors. First, the presence and severity of spasticity likely influences upper limb positioning during gait, with more affected sides showing greater deviations from normal patterns. Second, improved lower extremity alignment and function after surgery may lead to better trunk control and balance reactions, subsequently affecting upper limb positioning. It’s important to note that our findings are specific to children with spastic CP, and movement patterns in dyskinetic CP may differ significantly due to the distinct nature of motor dysfunction in these subtypes [15, 16].

According to the literature, advanced technologies such as Three-Dimensional (3D) gait analysis and wearable sensors are used in the evaluation of patients with CP in developed countries, while clinical examination and visual gait analysis have come to the fore in underdeveloped or developing countries due to the high cost of accessing these devices [9]. Visual gait analysis presents unique methodological challenges, particularly in the accurate measurement of joint angles. Beyond parallax errors, a critical consideration is the potential measurement inaccuracy caused by segment rotation. When measuring angles from 2D video frames, both body segments must lie precisely in the intended plane of measurement (sagittal or frontal). Any rotation of the arm or leg out of this plane can result in significant measurement errors that cannot be corrected through simple parallax adjustment [17]. This limitation is particularly relevant for upper extremity analysis, where arm rotation is common during gait.

 The biomechanical connection between upper and lower extremities during gait has been well documented in recent literature [18, 19]. Our findings support this relationship, demonstrating that surgical correction of lower extremity alignment can lead to measurable changes in upper limb kinematics. The clinical significance of these changes is particularly evident in the improvement of elbow flexion angles during midstance, which showed a mean reduction from 54.87° to 20.53°, approaching values seen in typically developing children. This improvement suggests enhanced dynamic stability during gait and potentially more efficient energy expenditure [20].

Due to the reliability of the Kinovea application, which is a simple software developed to reduce the cost of 3D gait analysis, Gonzalez et al. examined the intraobserver and interobserver reliability of Kinovea and its compatibility with Three-Dimensional (3D) gait analysis [21]. The ICCs were above 0.9, as in our study, with an intraobserver margin of error of 2.5° and an interobserver margin of error of 5° [21]. There are also studies [21–23] that support our finding that Kinovea can be used for gait analysis and kinematic measurements.

Gait in children with CP is a complex of movements of the lower extremities, upper extremities, trunk, and head and should be considered as a whole [19]. Studies have shown that children with CP develop a postural strategy in which the head and trunk move as a single segment, causing the entire body to sway from left to right [24]. Head and trunk movements are influenced by biomechanical connections within the tight kinematic chain of the thorax. One study suggested that the thorax of children with CP has a significantly greater Range of Motion (ROM) in the anterior plane than that of children with TD [25]. This suggests that changes in medial and lateral deflections of the head and trunk may be a consequence of changes in thoracic kinematics. In support of these findings, the results of two previous studies by Summa et al. [26] and Bonnefoy-Mazure [8] concluded that children with CP walk with significantly greater thoracic tilt and obliquity than do TD children. A segmental analysis of trunk kinematics by Attias et al. showed that the thorax, trunk, and upper limbs are biomechanically connected segments that may play an important role in gait characteristics [27]. A review by Hazari et al. revealed a significant difference in head and trunk kinetics and kinematics between children with CP and age-matched TD children [28]. While our trunk and head movement measurements showed some inconsistency with these previous findings, this may be attributed to the inherent challenges in accurately measuring these larger body segments using 2D video analysis, where even small rotational deviations can significantly impact angle measurements.

### Study limitations

Considering the limitations of our study, the inclusion of diplegic and hemiplegic patients may have caused bias. Bilateral upper and lower limb involvement was observed in the diplegic group, whereas hemiplegic patients had unilateral involvement. Consequently, the clinical course and treatment of these patients may differ. A significant methodological limitation was the inherent constraint of 2D video analysis in capturing complex three-dimensional movements. While we attempted to minimize measurement errors by capturing multiple camera angles and using high-quality video processing, the challenge of accurate angle measurement when body segments are not perfectly aligned with the capture plane remains a fundamental limitation of this approach. The margin of error of up to 2.5-5 degrees in the measurements made on Kinovea and the increase in margin of error when taking the midpoint in large joints, such as the trunk and head, were also limiting factors. We acknowledge that the inclusion of multiple surgical interventions may introduce heterogeneity into the outcomes, which represents a limitation of the study. However, the aim was to evaluate the general impact of lower extremity surgery on upper limb kinematics rather than specific procedures.

Another important limitation of our study is the high exclusion rate, with only 30 out of 300 screened patients meeting all inclusion criteria. The majority of exclusions were due to technical issues such as inadequate video quality, missing pre- or postoperative gait recordings, incompatible file formats, or the use of orthotics during gait — challenges inherent to retrospective video-based gait analysis. While these stringent criteria ensured high measurement accuracy and reliability, they inevitably reduced the sample size and may have introduced selection bias. Furthermore, the single-center design of this study may limit the external validity of our findings. Therefore, caution should be exercised when generalizing these results to the broader population of children with CP, particularly those treated in different clinical settings or with varying access to gait analysis facilities.

## Future

This retrospective comparative study evaluated upper extremity kinematics after lower extremity surgery and demonstrated changes. Conducting this study prospectively and in larger patient groups using 3D analysis and comparing the results will lead to more reliable results. If changes in the upper extremity are studied in more detail with additional studies, we anticipate that parameters to evaluate the upper extremity can be added to the EVGS scale. Our findings suggest that lower extremity surgery not only improves lower limb function but also influences upper extremity kinematics, highlighting the importance of considering the whole-body impact of surgical interventions. Future research should focus on understanding the mechanisms behind these improvements and their implications for surgical planning and rehabilitation strategies.

## Conclusion

Our study demonstrated that lower extremity surgery in children with cerebral palsy leads to significant improvements in upper extremity kinematics, with both affected and unaffected sides showing measurable changes in wrist and elbow positioning during gait. These findings have important clinical implications: First, they suggest that surgical planning for lower extremity procedures should consider potential effects on upper body movements. Second, the improvements we observed in upper extremity kinematics may contribute to better overall gait efficiency and functional outcomes. Third, our results support the concept of integrated treatment approaches that consider the interconnected nature of upper and lower body function in CP. This research has also improved the assessment of upper limb kinematics by providing a new method of measurement for clinics performing observational analyses in patients with CP. Furthermore, these findings could influence post-surgical rehabilitation protocols, suggesting the need for therapeutic approaches that address both upper and lower body coordination. Although the retrospective nature of this study had some limitations, these findings highlight the need for further prospective studies to deepen our understanding and confirm these results.

## Data Availability

The datasets generated and/or analyzed during the current study are not publicly available but are available from the corresponding author upon reasonable request.

## Abbreviations

CP: Cerebral palsy
TD: Typically developed
EVGS: Edinburgh visual gait score
3D: Three-dimensional
2D: Two-dimensional
ICC: Intraclass correlation coefficient
ROM: Range of motion
CI: Confidence interval
MA: More affected
LA: Less affected
